# Effect of Leukoreduction on Hematobiochemical Parameters and Storage Hemolysis in Canine Whole Blood Units

**DOI:** 10.3390/ani11040925

**Published:** 2021-03-24

**Authors:** Maria Teresa Antognoni, Maria Luisa Marenzoni, Ambra Lisa Misia, Luca Avellini, Elisabetta Chiaradia, Alessandra Gavazza, Arianna Miglio

**Affiliations:** 1 Department of Veterinary Medicine, University of Perugia, Via San Costanzo 4, 06126 Perugia, Italy; maria.antognoni@unipg.it (M.T.A.); marialuisa.marenzoni@unipg.it (M.L.M.); ambralisa@hotmail.it (A.L.M.); luca.avellini@unipg.it (L.A.); elisabetta.chiaradia@unipg.it (E.C.); 2 School of Bioscences and Veterinary Medicine, University of Camerino, 62024 Camerino, Italy; alessandra.gavazza@unicam.it

**Keywords:** storage lesions, whole blood units, blood transfusion, leukoreduction, dog

## Abstract

**Simple Summary:**

During the storage of blood units, cells undergo many changes, defined as storage lesions; these are biochemical, morphological and immunological modifications and seem to be responsible for adverse post-transfusion effects in recipients. The pre-storage leukoreduction seems to reduce them. The aims of this study are both to evaluate the human filter effectiveness and the effect of pre-storage leukoreduction in stored canine whole blood units. We tested whole blood units, leukoreduced and not, obtained from seven enrolled subjects, until the 42nd day. The white blood cell (WBC) and platelet (PLT) counts are reported to express the leukoreduction effectiveness. As indicators of storage-induced hemolysis, the lactate dehydrogenase activity (LDH) and sodium, potassium, and chlorine electrolytes were measured in plasma, and the red blood cell (RBC) count, hemoglobin concentration (Hgb), and hematocrit (Hct) were obtained with the complete blood count (CBC). The mean corpuscular volume (MCV) values and morphological index obtained from blood smear evaluation were used as indices of morphological changes. We observed that the leukoreduction filter for human use is equally effective on canine whole blood and that leukoreduction has a partially protective role to prevent some storage lesions.

**Abstract:**

Storage lesions (SLs) occur when the red blood cell quality is altered during the preservation of blood units. Pre-storage leukoreduction would limit the number of SLs. The aims of this study were to evaluate the effectiveness of a leukoreduction filter for human use and the effect of pre-storage leukoreduction on some ematobiochemical parameters in stored canine whole blood. Seven canine blood units were tested. Each one was divided into two units—one leukoreduced (LRWB) and one non-leukoreduced (nLRWB). On each unit, we determined the complete blood count (CBC), lactate-dehydrogenase (LDH), electrolytes (Na^+^, K^+^, Cl^−^), morphological index (MI) and hemolysis, on storage days 0, 7, 14, 21, 28, 35, and 42. Leukoreduction allowed a 98.30% recovery of the RBC count, retaining 99.69% and 94.91% of WBCs and PLTs, respectively. We detected a significant increase of LDH and MI with strongly higher values in nLRWB compared to LRWB. A progressive increase in electrolytes and LDH concentrations was observed as indices of stored hemolysis. LDH showed significantly lower values in LRWB units compared to nLRWB, suggesting its release from leukocytes. In the majority of units, hemolysis reached 1% on the 42nd day of storage. We assert the human leukoreduction filter effectiveness on canine whole blood, and we recommend using nLRWB before day 14, especially for critically ill patients. The difference of the basal hemolysis (day 0) percentages observed between subjects suggests that more studies should be performed to confirm a possible inter-individual donor biological variability of RBC membrane resistance, as happens in humans.

## 1. Introduction

Blood transfusion represents an essential therapeutic intervention in some hematological diseases and in emergency critical care. It is important that transfused red blood cells (RBCs) preserve their metabolic capacity and mechanical functions [1]. In recent years, the interest in this field of veterinary medicine has grown exponentially and the ability to store blood for a long time has profoundly revolutionized transfusion medicine. A blood bank allows the immediate availability of whole blood and blood components, and this translates into a timelier intervention in all those situations in which it is necessary to transfuse patients within a short time. Blood bags can be stored for a period of time between 35 (whole blood units—WB) and 42 (packed red blood cells—pRBC) days depending on the product [2,3]. To date, the studies carried out have been aimed at finding the best storage conditions through the addition of appropriate maintenance solutions, in order to extend the storage times and maintain the stored cells’ viability and functionality [4]. However, based on recent studies, it has been demonstrated that blood undergoes many changes during storage (storage lesions—SLs). SLs are responsible for negative post-transfusion effects in recipients, particularly in critically ill patients, and are classified into three categories: biochemical, biomechanical or morphological, and immunological changes [4]. Particularly, biochemical changes include the increase of potassium and sodium and hemolysis and oxidative injury [1,2,5,6,7,8]; biomechanical or morphological modifications include shape alterations and a reduction of RBC deformability. The modifications related to the corpuscular components of blood units could relate to RBC, white blood cells (WBCs) and platelets (PLTs). Those related to RBCs are oxidative injury [7], hemolysis [2,6], morphological alterations and changes in membrane composition [1], changes in sodium and potassium levels [1,5,8], and the release of procoagulant microparticles [9,10]; those connected to WBCs and PLTs are the increase in interleukin (Il-8) and VEGF [11,12,13], and the release of procoagulant phospholipid (PPL) [14] and microparticles [15].

For both humans and pets, it appears that the risks of harm associated with transfusions increase with the increase in the storage time [16,17,18] and in the number of transfused units to the same recipient [19,20]. However, it is difficult to unequivocally establish a cause-and-effect relationship between the transfusion of stored blood units and the negative clinical effects observed in transfused patients, since this could be due both to a specific storage lesion and to the severity of the disease [21].

Among SLs, hemolysis and those lesions caused by procoagulant and proinflammatory factors released by WBCs and PLTs appear to have great soundness. Hemolysis is one of the most studied modifications during blood storage and it is caused by the alteration of the erythrocytes’ shape as well as the increase in their fragility due to oxidative damage and modification of membrane proteins during storage. These changes seem to be particularly dangerous when blood units are transfused in an emergency, increasing the risk of infection and mortality of patients [19,22,23,24]. It has been documented that the practice of pre-storage leukoreduction, a process by which WBCs and PLTs are physically removed from whole blood units before it is processed in the various blood components, seems to have an important role on the maintenance of blood quality. [25]. In human medicine, this practice is now routinely used, while it is uncommon in the veterinary field [25]. It seems that leukoreduction can help partially reduce the effects of SLs [14,25]. Since WBCs are metabolically active cells that produce inflammatory cytokines, leukoreduction would be a benefit reducing the immunological storage lesions that induce an inflammatory reaction in the recipient [13,26] [13,27,28,29]. It has been shown that the leukocyte “contamination” in stored whole blood and its derivatives has been both associated with the presence of post-transfusion adverse reactions (non-hemolytic febrile reactions, alloimmunization, immunosuppression, transmission of pathogens, thromboembolic damage, and pulmonary micro-embolism) [13,26]. In veterinary medicine, little is known about the changes that occur during the storage of leukocyte-depleted blood units and the benefit associated with the practice of leukoreduction should be deeply studied, also to justify its relatively high economic costs [25].

The aims of our study were to evaluate the efficacy of a filter system used in the human field on canine whole blood and to document the storage effect in some hematological (RBC, Hgb, Hct, WBC, PLT), biochemical (LDH, electrolytes), and functional parameters (storage hemolysis) in stored leukoreduced whole blood compared to non-leukoreduced whole blood, given the need to increase knowledge on this blood product commonly used in emergency critical care.

## 2. Materials and Methods

### 2.1. Blood Donors

Blood units were obtained from seven Ariégeois dogs belonging to the volunteer donors program of the Veterinary Blood Bank (EMOVET-UNIPG) of the Department of Veterinary Medicine, University of Perugia, Italy. The animals met the following requirements for blood donors according to the Italian Ministry of Health guidelines [30,31,32,33,34,35]: age two to eight years, weight > 25 kg, regular vaccination status, and regular prophylaxis against ecto- and endoparasites. The state of health was established on the basis of the anamnesis, clinical examination, CBC (complete blood count), and serum biochemistry panel. Serological tests have been performed to exclude haematological infections: *Leishmania infantum*, *Ehrlichia canis*, *Babesia* spp., *Rickettsia* spp., *Anaplasma phagocytophilum*, and *Dirofilaria immitis*.

### 2.2. Blood Collection

Blood was collected according to the Italian veterinary transfusion guidelines [30,31,32,33,36]. The collection was performed by venipuncture from the jugular vein after trichotomy, cleansing and disinfection of the area; no sedation was performed. A unit of 350 mL of WB was taken from each subject, using a commercial closed collection system for human use, approved by the Italian Ministry of Health. It consisted of a quadruple bag made of PVC (Fenwal Inc., Lake Zurich, IL 60047, USA), containing 49 mL of CPDA1 anticoagulant-preservative solution and was equipped with an integrated filter for pre-storage leukoreduction (Kansuk laboratori: besyol Mah. Eski Londra asfalti, No 4, 34620 Sefakoy/Istanbul; Pall, WBF3 leukocyte filter for whole blood). The bags were attached to the filter system using a sterile technique and filtered through the leukoreduction filter into a secondary bag by gravity after breakage of an integral canal above the filter. The leukoreduction filter used in this study is a third-generation polyurethane filter with a neutral charge, manufactured to remove both WBCs and PLTs. Immediately after collection, the “mother bag”, containing the collected blood, was weighed, and about half of its content were drained through the filter and collected by gravity in the satellite bag. This procedure allowed to obtain approximately 175 mL of non-leukoreduced WB (nLRWB) and 175 mL of leukoreduced WB (LRWB) for each unit. A total of seven LRWB units and seven nLRWB units were tested. Each unit was stored in a blood bank refrigerator at 3 to 4 °C (SANYO Blood BankRefrigerator, model MBR-107D (H)), for 42 days and mixed gently at least once a day.

### 2.3. Sample Analysis

Three aliquots (A: 10 mL, B: 7 mL, C: 5 mL) of blood were aseptically obtained from all the bags (nLRWB and LRWB) on storage days 0 (T0, day of donation), 7 (T1), 14 (T2), 21 (T3), 28 (T4), 35 (T5), and 42 (T6) after having appropriately suspended the corpuscular part. Sampling was carried out under a laminar flow hood to ensure sterility conditions. These aliquots were collected in an anticoagulant-free tube.

In vitro analysis, evaluated from the aliquots A, B, and C at each time point, were the following:

- A: CBC, % of WBC and PLT depletion to evaluate leukoreduction effectiveness, % RBC recovery after filtration (only at T0), LDH, electrolytes concentrations, and blood smear evaluation.
- B: % of storage hemolysis.
- C: aerobic and anaerobic bacterial culture.

#### 2.3.1. Complete Blood Count

CBC were obtained with an automated laser analyzer (Sysmex-XT1800iV; Sysmex, Kobe, Japan) and the following parameters were assessed in nLRWB an LRWB units for each time point: RBC (n. × 10^6^/μL), Hgb (g/dL), Hct (%), MCV (fL), RDW (%), WBC (n. × 10^3^/μL), PLT (n. × 10^3^/μL).

#### 2.3.2. Percentages of WBC and PLT Depletion (Leukoreduction Effectiveness)

To calculate the effectiveness of filtration, the WBC % mean and the PLT % mean at T0 was estimated both in LRWB and in nLRWB units. The following formula was used to determine the percentage of leukoreduction as previously established [6]:

%WBC depletion = (WBC mean in nLRWB − WBC mean in LRWB) × 100/WBC mean in nLRWB(1)

%PLT depletion = (PLT mean in nLRWB − PLT mean in LRWB) × 100/PLT mean in nLRWB(2)

#### 2.3.3. Percentage of RBC Recovery

To calculate the effectiveness of RBC recovery after filtration, the RBC mean at T0 was estimated both in LRWB and nLRWB units. The following formula was used to determine the percentage of RBC recovery as previously established [6]:

%RBC recovery = (RBC mean in nLRWB − RBC mean in LRWB) × 100/RBC mean in nLRWB(3)

#### 2.3.4. LDH and Electrolytes

After the blood counts, samples were centrifuged (3000× *g* for 10 min-ALC CENTRIFUGE PK120) and the supernatant was used to evaluate the concentrations of LDH (U/L) by using an automatic biochemistry analyser (Hitachi-904 Boehringer, Mannheim, Germany), and to determine the concentrations of Na^+^, K^+^ and Cl^−^ (mEq/L) with an electrode analyzer (i-smart 30 VET, FUTURLAB, Limena, (PD) Italy).

#### 2.3.5. Blood Smear Evaluation and Morphological Index

Each blood smear performed was stained with May Grünwald–Giemsa stain by an automatic slide-stainer (Wescor Aerospray slide stainer, 7120. Delcon, Bergamo, Italy). The microscopic evaluation of each smear was carried out by the same hemathologist (M.T.A.) using a standardized method. The slides were observed under an optical microscope, before at 200× objective magnification for cellularity assessment, and then at 1000× objective magnification for RBC morphology evaluation. A total of 200 RBCs were counted and classified, based on morphology of cells, and a score from 0 to +3 was assigned in order to calculate the morphological index (MI) [29,37,38]:

- 0: Discocyte (normal form erythrocyte)
- 1: Echinocyte I (irregularly shaped erythrocyte, with a maximum of five membrane spicules)
- 2: Echinocyte II (flat erythrocyte with numerous membranous spicules)
- 3: Echinocyte III (ovoid or spherical erythrocyte with numerous membranous spicules)

At all time points, both from the LRWB and nLRWB units, for each specimen the morphological index (MI) was calculated with the following formula (MI = ∑ scores/200) as previously described [29]. Subsequently, the mean of MI values at each time points, both in LRWB and in nLRWB units, was calculated.

#### 2.3.6. Storage Hemolysis

The percentages of hemolysis in all stored LRWB and nLRWB units at each time point (T0 = basal hemolysis, T1–T6 = storage hemolysis) was calculated using the following formula [5]:

% hemolysis (storage hemolysis) = [(100 − Hct) × supernatant Hb]/total Hb(4)

The Hct and total Hb concentration were obtained from the CBC.

Supernatants were obtained on plasma, separated by centrifugation (2000× *g* for 10 min, Eppendorf-5810 R) of 1 mL of blood from each B aliquot. The concentrations of supernatant Hb were measured using the direct spectrophotometric method (HewlettPackard 8452A spectrophotometer) as the Harboe assay previously described [39], with appropriate correction, according to the following formula [40].

Supernatant Hb = [(167.2 × A_415nm_) − (83.6 × A_380nm_) − (83.6 × A_450nm_)] × 1/1000 (5)

#### 2.3.7. Bacterial Culture

To exclude bacterial contamination, from each C aliquot obtained both in LRWB and nLRWB, at each time point, aerobic and anaerobic bacterial culture was carried out as previously described [31,33,35]. Tryptic soy broth (10 mL) medium was inoculated with each sample and incubated aerobically and anaerobically at 37 °C for 48 h. After 48 h, a small amount of each broth culture was subcultered on blood agar, MacConkey agar, and mannitol salt agar and incubated for 48 h at 37 °C.

### 2.4. Statistical Analysis

A general linear model for repeated measures was used to evaluate differences between groups (LRWB and nLRWB) and variables with repeated measurements (HCT, RBC, Hgb, MCV, RDW, MCH, MCHC, WBC, PLT, K, Cl, Na, LDH, MI). The Bonferroni post-hoc test was applied to identify differences in times of the variables with repeated measurements. Comparison between basal hemolysis of the LRWB and nLRWB was performed by a paired *t*-test. Data were analyzed by commercial software R, version 2.8.1 (R, Development Core Team 2007). A value of *p* < 0.05 was considered significant for the analysis. The Shapiro–Wilk test was used for the evaluation of normality.

## 3. Results

### 3.1. Complete Blood Count

Results regarding all parameters included the complete blood count in nLRWB and LRWB during times are reported in Table 1.

### 3.2. Percentages of WBC and PLT Depletion, and Percentage of RBC Recovery (Leukoreduction Effectiveness)

**Figure 1:**
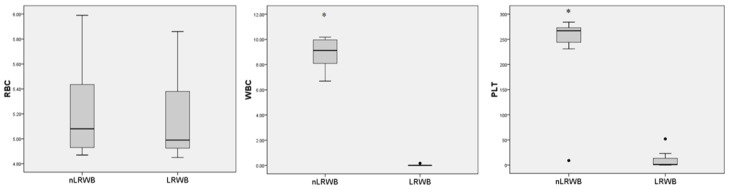
Leukoreduction effectiveness. Box plots (indicating median as lane and interquartile range as box) of RBC(×10^12^/L), WBC(×10^9^/L), and PLT(×10^9^/L) counts before (nLRWB) and after (LRWB) leukoreduction of whole blood units from seven canine donors. Black circles indicate outliers. Asterisks * *p* < 0.05.

The pre-storage leukoreduction allowed to obtain a reduction of WBCs and PLTs of 99.69% and 94.91%, respectively, and in the units treated with leukoreduction the RBC recovery percentage was 98.30% (Figure 1).

**Figure 2:**
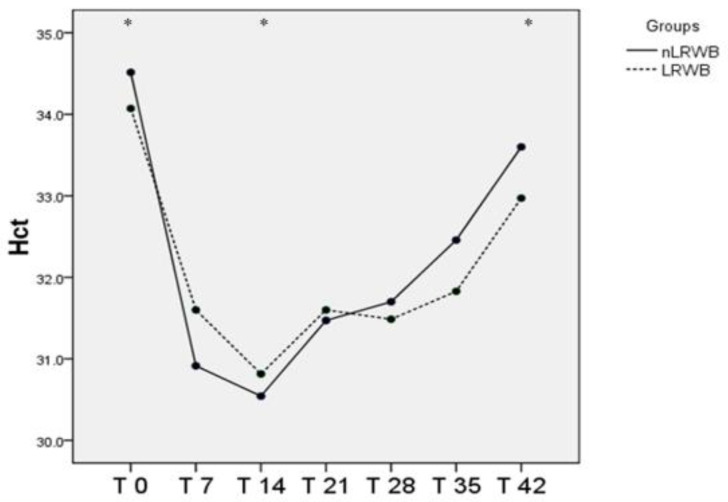
Differences in Hct (mean) measured in nLRWB and LRWB units during the storage period (*p* < 0.05). The six weeks of storage are presented in the x-axis; the T0 corresponds with the day of donation (day 0). Hct (%) is presented in the y-axis. Asterisks * *p* < 0.05.

The WB and the PLT counts measured in nLRWB units during the storage time have no significant differences. The RBC and Hb values measured in all units during the storage period remained substantially unchanged, with no significative differences between LRWB and nLRWB units and among the different storage times (Table 1). The Hct showed statistically significantly modifications during storage (Table 1, Figure 2), but without significant differences between LRWB and nLRWB units. There was a decrease from T0 to T14 and a progressive increase from T14 to T42 in all units, without significant differences between LRBW and nLRWB.

**Figure 3:**
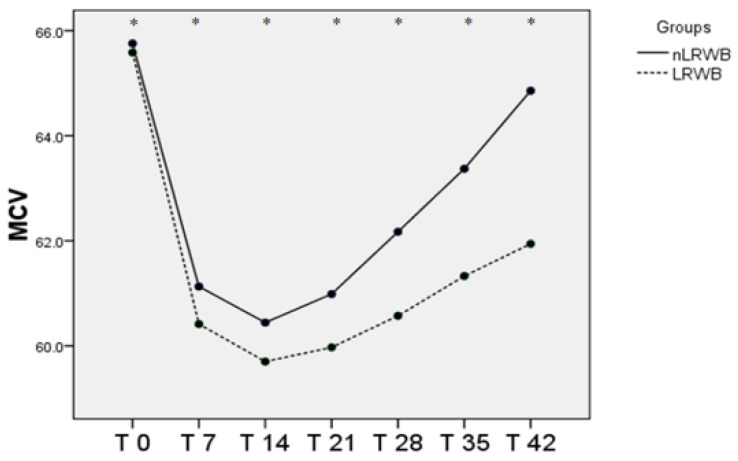
Differences in MCV (mean) measured in nLRWB and LRWB units during the storage period. The days of storage are presented in the x-axis. MCV (fL) is presented in the y-axis. Asterisks * *p* < 0.05.

The MCV shows significant differences in almost all the times considered, except T0, without significant differences in LRWB and nLRWB units, as shown in Table 1 and Figure 3 After a reduction from T0 toT7, there is a progressive increase from T14 toT42.

**Figure 4:**
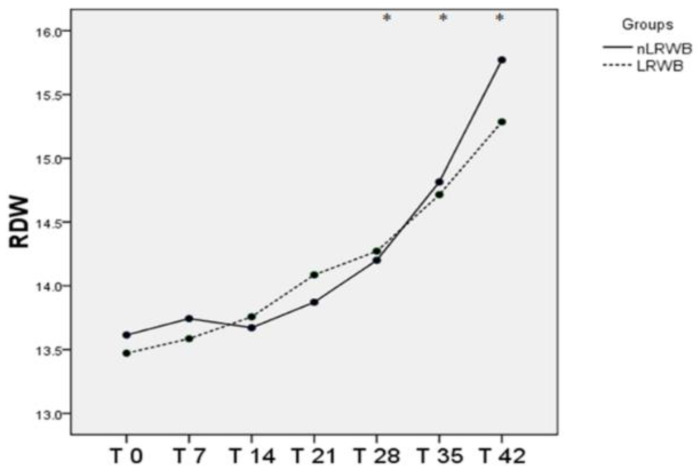
Differences in RDW (mean) measured in nLRWB and LRWB units during the storage period. The days of storage are presented in the x-axis. RDW (%) is presented in the y-axis. Asterisks * *p* < 0.05.

The RDW increases during the storage in both groups, in particular, significantly from T28 to T42 with no differences between LRWB and nLRWB units (Table 1, Figure 4).

### 3.3. LDH and Electrolytes

Results regarding LDH and electrolyte levels during times and among groups are reported in Table 2.

**Figure 5:**
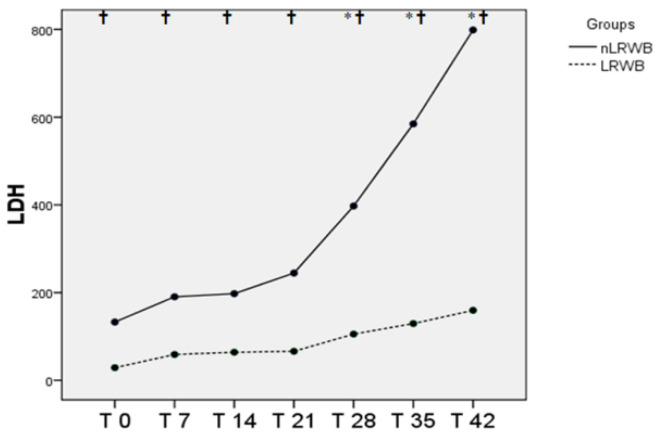
Differences in LDH (mean) measured in nLRWB and LRWB units during the storage period. The days of storage are presented in the x-axis. LDH (U/L) is presented in the y-axis. Asterisks * *p* < 0.05.

The changes in plasma concentration of LDH enzyme are shown in Figure 5 There are significant differences both between the LRWB and nLRWB groups, and among all the storage times, with a progressive increase in LDH levels during the six weeks of storage, more marked starting from T21.

**Figure 6:**
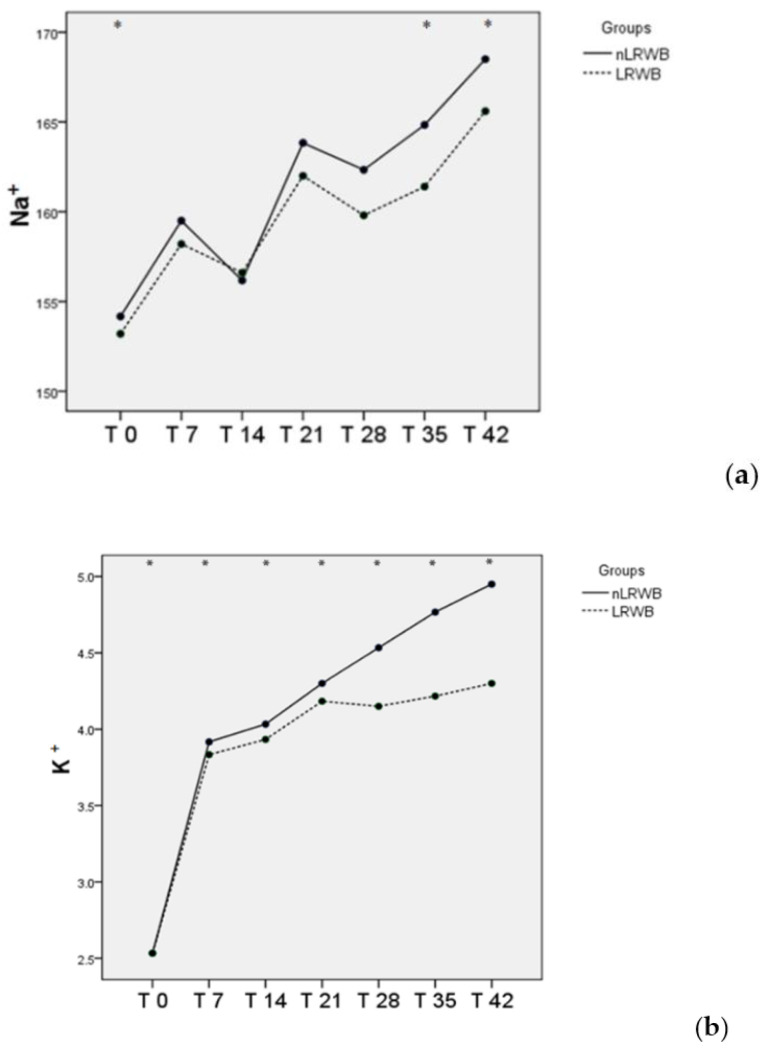
Differences in Na^+^ (mean) (**a**) and K^+^ (mean) (**b**) measured in nLRWB and LRWB units, during the storage period. The days of storage are presented in the x-axis. Na^+^ and K^+^(mEq/L) is presented in the y-axis. Asterisks * *p* < 0.05.

No significant differences are observed between the LRWB and nLRWB units regarding Na^+^ and K^+^. Na^+^ showed a progressive increase from T0 to T42, except at T14 and T28, which showed a reduction. T0 is significantly different from all storage times (Figure 6a). The mean K^+^ concentration showed a progressive increase, particularly from T0 to T7. From T21 to T42, the differences between LRWB and nLRWB increased, but not significantly (Figure 6b).

**Figure 7:**
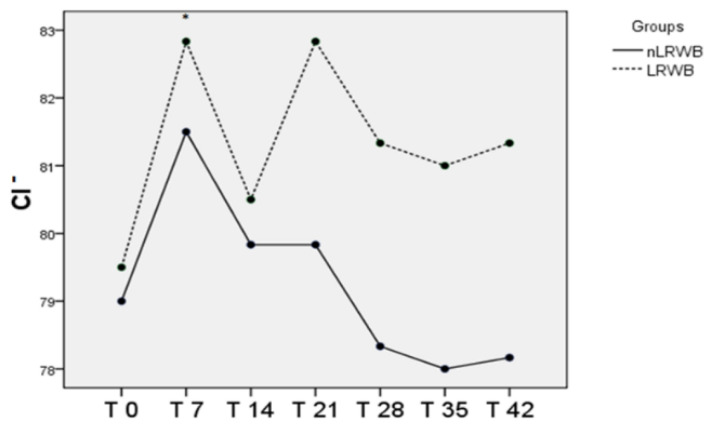
Differences in Cl^−^ (mean) measured in nLRWB and LRWB units, during the storage period. The days of storage are presented in the x-axis. Cl^−^(mEq/L) is presented in the y-axis. Asterisks * *p* < 0.05.

The Cl^−^ concentration showed a more variable trend, alternating increasing and decreasing values, and then acquired an almost stationary phase from T21. There are no differences between the LRWB and nLRWB units (Figure 7).

### 3.4. Blood Smear Evaluation and Morphological Index

**Figure 8:**
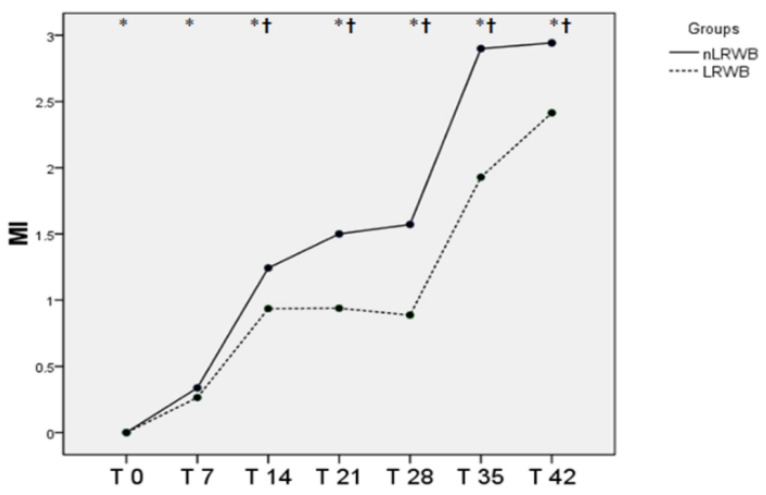
Differences in morphological index (mean) measured in nLRWB and LRWB units, during the storage period. The days of storage are presented in the x-axis. MI is presented in the y-axis. Asterisks * *p* < 0.05.

**Figure 9:**
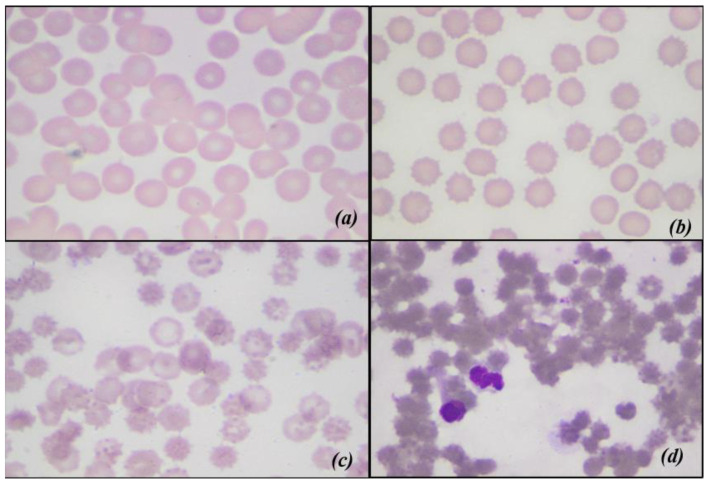
Optical microscopic scans of non-leukoreduced whole blood, showing morphological changes during storage. (**a**) corresponds to T0 red blood cells with normal biconcave disc shape; (**b**) corresponds to T14; (**c**) correspond to T28 and (**d**) corresponds to T42 progressive changes in shape. (**d**) also shows neutrophils in the degeneration phase.

**Figure 10:**
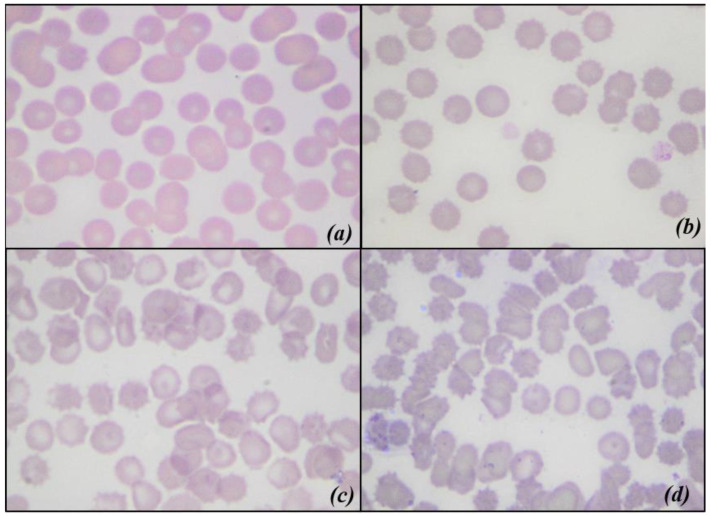
Optical microscopic scans of leukoreduced whole blood showing morphological changes during storage. (**a**) corresponds to T0 red blood cells with normal biconcave disc shape; (**b**) corresponds to T14; (**c**) correspond to T28 and (**d**) corresponds to T42 progressive changes in shape.

The changes in MI are shown in Figure 8 There is a significant difference between the whole units and the filtrates, and the times are all different from each other. Below, there are some of the images obtained with an optical microscope, from blood smears of nLRWB (Figure 9) and LRWB (Figure 10), which show the progressive transformation of the red blood cell from discocyte to echinocyte during the storage time.

### 3.5. Basal and Storage Hemolysis

The percentages of hemolysis at T0 (basal hemolysis) showed an interindividual variability between subjects, although this was not significant.

**Figure 11:**
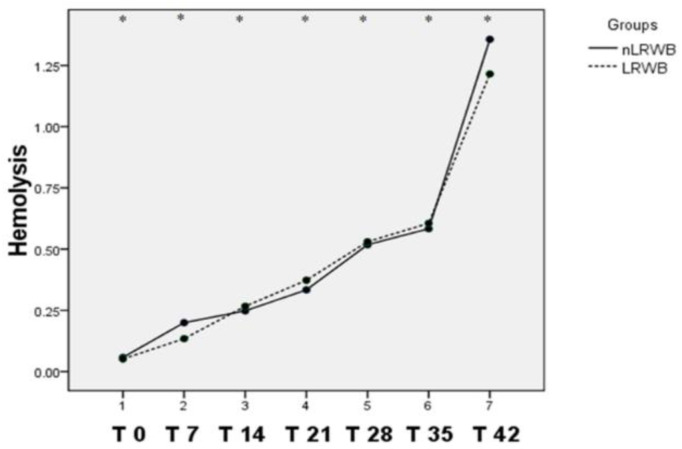
Basal and storage hemolysis. Differences in hemolysis (mean) measured in nLRWB and LRWB units during the storage period. The days of storage are presented in the x-axis. Hemolysis (%) is presented in the y-axis. Asterisks * *p* < 0.05.

The degree of hemolysis showed progressively significant increases from T0 to T42 in both the nLRWB and LRWB units (Figure 11). There are no significant differences in the percentages of storage hemolysis manifested by the whole units compared to the leukoreduced ones.

### 3.6. Bacterial Culture

All samples for aerobic and anaerobic bacterial culture were negative.

## 4. Discussion

The interest in leukocyte-depleted whole blood and packed red blood cell transfusion has increased in recent years in order to minimize the risk in the transfusion recipient. In the human field, it has been established that the removal of buffy coat from the whole blood allows the elimination of about 70% of the WBCs, and it seems to be sufficient in preventing febrile transfusion reactions due to blood transfusion but not to prevent a possible alloimmunization secondary to the human leukocyte antigen system (HLA) [41]. Filtration has recently emerged as the most commonly used method of leukocyte depletion. Leukocyte reduction by means of filtration can be performed before and after storage. Prestorage leukofiltration offers additional benefits, avoiding the potential of reaction and alloimmunization compared to leukocyte removal during or after the storage period [41]. Particularly, HLA antigens may solubilize from leukocyte membranes during storage, passing through the filter used after storage and immunizing the recipient [41,42,43]; cytokines released from leukocytes during storage may cause febrile reactions in transfusion recipients [44,45] and the release of leukocyte enzymes during storage may alter RBC metabolism and reduce their half-life [41,46].

The results of our study are encouraging since significant changes in some specific parameters have been found both between leukoreduced and non-leukoreduced whole blood units during storage time. We decided to evaluate these parameters for a period of time of a week longer (42 days) than the normal shelf-life commonly stated for whole blood units (35 days), to better evaluate their modifications during storage.

The leukoreduction filters currently available are specific for human use. A unit of whole blood generally contains ≥1 to 10 × 10^9^ WBC. In human transfusion medicine, the Food and Drug Administration (FDA) establishes that a blood component can be defined as leukoreduced when the concentration of white blood cells is <5.0 × 10^6^ with 85% recovery of original RBC content [47] and <1 × 10^6^ WBC/unit is the standard of the Council of Europe [3,48]. In our study, leukoreduction was effective since filtration was under the FDA standards, removing 99.69% leukocytes and 94.91% platelets from the whole blood units and ensuring the 98.30% RBC recovery in LRWB units compared to the nLRWB units. Our results are in agreement with those previously found on canine leukoreduced packed red blood cell units and whole blood units [6,11,49] using a filter currently available for human use. Our study confirms that the use of commercially available leukoreduction filters for human blood units is effective also for canine whole blood.

Regarding the hematological parameters studied, Hb maintained similar values over time, similar to other studies [1,2]. Indeed, we found that Hb concentrations were not statistically different from the beginning to the end of the storage time both in LRWB and nLRWB units. In our study, as well as in the above-mentioned studies (1,2), we detected the total hemoglobin levels including both the intracellular and extracellular Hb content. In human medicine, some authors measured only plasma Hb in blood units by using a specific instrument able to detect low plasma Hb levels and found a statistically significant increase during storage of this parameter, which can be used as a more reliable index of hemolysis [7]. Unfortunately, we could not obtain this type of determination.

The Hct values varied significantly during the storage time, both in LRWB and in nLRWB units, showing a decrease on the 14th day (T2) of storage compared to T0 levels and an increase on the 42nd day (T5), returning to basal levels. No significant differences were found between the LRWB and nLRWB units, probably because leukoreduction does not affect this parameter. Our results are different to those previously found in packed red blood cell units both in human [50] and veterinary medicine [2] that found a progressively significant increase in Hct from the beginning to the end of the storage time (42nd day). A possible explanation for this difference in our study could be the different effect of plasma in whole blood units compared to the maintenance storage solutions used (Adsol, Optisol, SAGM, and PAGGGM) in packed red blood cell units of the previous studies [2,9]. Another possible explanation for the differing results could also be due to the different methods used to obtain the Hct value. In the previous research [2], Hct was directly measured using the microhematocrit method, whereas in the present study we used an automated hematology analyzer that indirectly determined Hct values by multiplying RBC numbers with the MCV. In our study, the RBC count remained unchanged during storage and the MCV showed a trend similar to that of Hct (a significant decrease at the 14th day and a significant increase, reaching the basal levels, at the 35th and 42nd days).

The MCV represents the average volume of erythrocytes and could be considered an index of morphological modification; its variability in all units during the six weeks of storage indicates the changes in size of red blood cells during storage. In our study, the MCV reaches higher values in nLRWB units in all time points. Moreover, MCV values showed a reduction at T2 (14 days) and then a progressive increase that reaches the basal levels at T5 (35 days) and T6 (42 days), both in the LRWB and nLRWB groups. The increase in the MCV values indicates RBC swelling; it seems that there is a deregulated mechanism of red blood cell volume. Particularly, it is documented that there is an insufficiency of the adenosine-5′-triphosphate (ATP)-dependent Na−K pumps of the RBC membrane which cannot work properly under cold storage conditions [33,48]. This could explain the increase in the volume of the RBC other than the hypocromia and the anisocytosis found [1]. Moreover, the increase in the MCV was more pronounced in unfiltered units, suggesting that some products of leukocyte disintegration may be involved to affect RBC membrane properties [33,48].

Our results are partly in contrast with those reported by other authors that record the slight increase of the MCV from T0 to T35 [48]. In our study, both MCV and Hct trends could be explained with the transformation of red blood cells into echinocytes starting from the 14th day of storage. In fact, with their numerous membrane projections, these modified erythrocytes would trap the plasma between the red blood cells for a value equal to 1 to 3% of the volume, as suggested by Mustafa et al. (2016) [50]. To confirm this theory, we evaluated blood smears and calculated the morphological index weekly from all the LRWB and nLRWB samples. We observed a progressive morphological change during the 42 days of storage with a progressive transformation of the discocyte, first into a flat echinocyte, then into a spherical echinocyte with numerous membrane spicules. Particularly, the 14th day of storage, when compared with day 0, shows a significant increase of MI, both in the nLRWB and in LRWB units. The MI progressively increases in both groups from T2 to T5, as does the MCV and Hct. In human [50] and veterinary medicine [38] it is assumed that the oxidative damage that occurs during storage could contribute to the morphological modifications. In our study, the severity of the morphological and MI changes were greater in the nLRWB units at all storage times (Figure 9). Since the entity of the increase was greater in nLRWB, we can suppose that the leukocyte degradation products could also contribute to the transformation of the red blood cell from discocyte to echinocyte. Our results are in agreement with those observed by other authors [29].

Furthermore, the increase of RDW values observed in our study during the storage period evaluated is an index of anisocytosis and deformation of the erythrocyte mass [29,39].

In our study, the degree of hemolysis during the storage time was calculated using the described formula, and indirectly by LDH and electrolytes. In human medicine, the length of blood storage time is regulated by the FDA, which has established that all commercially available preservative solutions must ensure the survival of 75% of the red blood cells after 24 h from the transfusion. The Council of Europe set the limit of hemolysis at 0.8% on the 42nd day of storage for packed red blood cells units [3], while the American Association of Blood Banks (AABB) admits a percentage of hemolysis not exceeding 1% [51,52,53,54]. There are no comparable guidelines about the acceptable hemolysis degree in stored canine whole blood [55].

In the present study, hemolysis was determined using the same formula applied in other human and veterinary studies [2,6,40,48,49,56]. In all the units, the degree of hemolysis progressively increased (*p* < 0.05) during the storage time, reaching the maximum level close to 1% at the 42nd day of storage (Figure 11). There are no significant differences in the percentage of hemolysis detected in the nLRWB whole blood units compared to the LRWB units. Only at the 42nd day of storage was the hemolysis percentage slightly higher in the nLRWB units. According to our results, leukoreduction did not have any evident effect on the percentage of hemolysis during storage. This practice would not seem to have a protective role on storage-induced hemolysis as would have been expected on the basis of what is reported in human literature [41,57]. Only a few human studies [56] show results similar to ours concerning the comparison of hemolysis percentage in leukoreduced and non-leukoreduced blood units. According to our knowledge, in the veterinary literature there is only one other study that compares the hemolysis percentage between leukoreduced and non-leukoreduced canine whole blood units [48].

In our study, hemolysis progressively increased during the storage time and our results are similar to those of other studies by using the same method for the hemolysis calculation, indicating a progressive increase in the hemolysis percentage during the storage time both in leukoreduced [6,49,56] and non-leukoreduced units [2,56]. Particularly, one study showed that the percentage of hemolysis increases independent of the storage solutions used (Adsol, Optisol, SAGM and PAGGGM) from day 0 to day 42 of storage [6]. Ferreira et al. (2018) [2] found a statistically significant positive correlation between the storage time and hemolysis in canine non-leukoreduced packed RBC stored for six weeks that exceeded the hemolysis limit of 1% in almost all of the units tested. Almost 51% of the units with 36 to 42 days of shelf-life showed more than 1% hemolysis. Interestingly, in our study, this limit was exceeded only at the 42nd day of storage in whole blood units in CPDA1, both in LRWB and in nLRWB, but with slightly higher values in nLRWB units. The use of whole blood units seems to be valid regarding the storage-induced hemolysis results in the veterinary practice.

It is worth mentioning that, in our study, there was a difference between the basal hemolysis percentages recorded in each subject on the donation day (T0). Indeed, as previously observed [58], the largest source of inter-unit difference in hemolysis is donor-specific. A donor-specific variability in RBC performance during storage, and post transfusion viability has been previously established [59,60]. The RBC lifespan and storage viability could be affected by numerous factors such as genetic variability (age, breed, gender), subclinical conditions, concentrations of membrane peroxiredoxin-2, the presence of serum uric acid, and lifestyle factors (diet, attitude) that cannot be revealed by the hematological profile obtained at blood donation. This donor variation effect seems to lead to the production of unequal blood products even in similar storage conditions and durations. [59,60]. More studies are needed in veterinary medicine regarding this topic, since few data are available.

To evaluate the degree of hemolysis during the storage time, other reliable hemolysis indexes such as LDH and electrolyte concentrations can also be used [61,62]. In our study, these parameters were significantly increased during the storage time. Indeed, a change in the plasma electrolyte concentration occured, certainly attributable to hemolysis, but also to alterations in the Na/K membrane pump. In our study, we found an increase in serum K^+^ and Na^+^ levels, from day T0 to T5 in both the LRWB and nLRWB groups, but with lower values in the LRWB group. Nevertheless, the K^+^ values remained close to the reference value during storage, while the Na^+^ values exceeded the higher reference limit. On the other hand, the Cl^−^ values showed limited significative differences between the LRWB and nLRWB units. Our results are in agreement with those reported in veterinary medicine by other authors that report a significant increase in plasma electrolytes, and in particular in K^+^ levels in canine WB and pRBC both in leukoreduced and non-leukoreduced whole blood units with different maintenance solutions added [5,48].

In human medicine, it is known that, during blood storage, a constant increase of plasma potassium that reaches high values and a decrease in plasma sodium levels occur [1,5,8]. The mechanisms underlying this electrolyte imbalance have been attributed to the reduced function of the Na/K-ATPase pump of the red blood cell membrane, causing the reduced entry of the sodium ions into the cells and a reduced exit of potassium ions from the cells via the semipermeable membrane. As previously mentioned, this pump works poorly during storage due to falling concentrations of ATP correlated to the cold storage conditions. At the same time, the potassium accumulated in the erythrocytes during storage is then subsequently released due to the increasing hemolysis [4,29,63,64].

The results of our study revealed significant alterations in the concentration of both potassium and sodium, whose concentrations increased in all units during the storage time, even if only the Na^+^ value exceeded the higher reference limit. These results are in contrast with those reported in the human literature [1,5,8]. Probably, this occurred because canine erythrocytes contain naturally lower potassium and higher sodium concentrations, in addition to having limited Na/K-ATPase pump activity, as compared to humans [65,66]. Furthermore, it has been documented that cation permeability is volume-dependent in canine erythrocytes. As a result, sodium permeability decreases, while potassium permeability increases as RBCs swell, and the opposite occurs as they shrink [65].

For these reasons in human medicine, particular attention is paid to recipients affected by coronary pathologies, subjected to cardiac bypass operations, as well as to pediatric patients [67]. The increase in K^+^ in the supernatant could lead to hyperkalemia in the recipient, with the risk of developing cardiac arrhythmias up to death from cardiac arrest. This can occur especially in the case of transfusions of red blood cells stored for a long period of time and performed quickly. On the other hand, in the preserved dog blood units, a significant increase in the concentration of K^+^ is unlikely to occur [4], as our results also demonstrate. However, some clinically healthy Japanese and Korean dog breeds show high levels of this electrolyte within the erythrocytes, resulting from a greater activity of the aforementioned pump [68] and a prolonged storage period of the blood units obtained from these breeds could lead to the accumulation of K^+^ in the supernatant over the reference limits [4].

In veterinary medicine, there are not many studies regarding modifications of the LDH concentrations during storage. According to our results, in all LRWB and nLRWB units, we found significant progressively increased LDH levels, with strongly lower values in LRWB bags. These changes are comparable to those found in human blood units [7,69,70], suggesting that LDH could be an index of reliable hemolysis also in canine blood bags. In our study, the increase was significantly higher in the nLRWB units compared to the LRWB groups according to what happens in human medicine [69]. These results confirm the progressive increase of hemolysis during the storage time, as previously stated with the storage hemolysis formula. Moreover, the evaluation of LDH concentrations in LRWB demonstrated that there is probably a presumptive protective role of leukodepletion. This result could be attributed to the LDH enzymatic activity contained in the white blood cells [71,72,73]. In addition, some authors affirm that the degradation of leukocytes causes the release of LDH, and other lytic enzymes which would alter the properties of the erythrocyte membrane responsible for further hemolysis during storage in nLRWB [74,75].

In the literature, different studies also justify the use of leukoreduction in the reduction of hemolysis during storage with various mechanisms. Some authors suggest that the leukoreduction filter could remove the less deformable and fragile red blood cells present at the time of collection [51]. Others [4,51,76] suggest that in nLWB units, WBCs contribute to the faster consumption of the energy substrates contained to produce ATP for their metabolism. This could cause a reduced ATP production by RBC that affects the erythrocyte membrane by reducing its elasticity and intracellular viscosity, with an increase in hemolysis.

The main limitations of this study are the small number of subjects enrolled, the impossibility of carrying out a statistical analysis of data regarding basal hemolysis related to the subject and the possible influence of genetic and lifestyle donor variability on blood parameters.

## 5. Conclusions

Based on the results of our study, we believe that the leukoreduction filter for human use is equally effective on canine whole blood units as it allowed a 98.30% recovery of RBC, retaining 99.69% and 94.91% of WBCs and PLTs, respectively.

A significant increase for the variables LDH and MI were detected among groups (nLRWB vs. LRWB), with higher values in non-leukoreduced units, demonstrating the usefulness of this practice in veterinary medicine. Moreover, a significant progressive increase of the parameters RDW, K^+^, Na^+^, LDH, and MI during T0 to T6 as well as a reduction of the parameters HCT and MCV at T2 (14 days) with their progressive increase that reaches the basal levels at T5 to T6 (35 days to 42 days) have been shown in both the LRWB and nLRWB groups.

Our results demonstrate that a storage time of 35 days is adequate for whole blood stored in CPDA1, as it is able to maintain the percentage of hemolysis below 1%, as required by human standards. However, given the progressive morphological changes shown during storage, already evident on day 14 on unfiltered samples, we recommend using LRWB stored units before the 14th day of storage, especially in critical and polytransfused patients.

We showed that the storage hemolysis increased significantly during storage time without significant differences between the LRWB and nLRWB units. This happened despite an insignificant difference observed for RBC levels during the storage period. Since the increase of LDH levels during storage time was different among the groups, we suppose that the change of its concentrations should not be correlated only with hemolysis. Probably, the highest levels of LDH recorded in the non-filtered samples could have a leukocyte origin. More studies are needed to confirm this data and to evaluate the post-transfusion effect of this enzyme in the recipients.

Finally, the detection of a difference between the basal hemolysis percentages recorded in each subject on the day of donation could suggest an inter-individual donor biological variability of RBC membrane resistance, as happens in humans. Further studies, aimed at evaluating the degree of hemolysis tendency of different blood donors are needed in the veterinary field, since no data are available. Particularly, future research should be conducted to define how much age, gender, race, diet, and other individual variables could influence the characteristics of the erythrocyte membrane.

## Figures and Tables

**Table 1:** Means ± standard deviations and significance of the parameters RBC(×10^12^/L), HCT(%), Hgb(g/dL), MCV(fL), RDW(%), WBC(×10^9^/L), PLT(×10^9^/L) in nLRWB and LRWB groups, in the different days post-donation (T0 to T6). nLRW: not leukoreduced whole blood, LRWB: leukoreduced whole blood.

Parameters	Groups	Day of Donation							*p* for Repeated Measurements	
		T0 Day 0	T1 Day 7	T2 Day 14	T3 Day 21	T4 Day 28	T5 Day 35	T6 Day 42	Among Times	Among Groups
RBC (×10^12^/L)	nLRWB	5.23 ± 0.422	5.05 ± 0.543	5.04 ± 0.552	5.15 ± 0.652	5.09 ± 0.656	5.11 ± 0.664	5.17 ± 0.676	0.85	0.97
	LRWB	5.18 ± 0.38	5.23 ± 0.36	5.16 ± 0.43	5.27 ± 0.426	5.19 ± 0.445	5.19 ± 0.474	5.32 ± 0.524		
HCT (%)	nLRWB	34.5 ± 4.1 *	30.9 ± 4.3	30.5 ±4.2 *	31.4 ± 4.7	31.7 ± 5.1	32.4 ± 5.2	33.6 ± 5.3 *	**<0.01** (*p* = 0.004)	0.96
	LRWB	34.0 ± 3.8 *	31.6 ± 2.8	30.8 ± 3.3 *	31.6 ± 3.2	31.4 ± 3.5	31.8 ± 3.7	32.9 ± 3.8 *		
Hb (g/dL)	nLRWB	12.5 ± 1.8	12.3 ± 2.1	12.3 ± 2.1	12.2 ± 2.2	12.2 ± 2.1	12.3 ± 2.1	12.3 ± 2.2	0.29	0.87
	LRWB	12.3 ± 1.7	12.5 ± 1.4	12.5 ± 1.5	12.5 ± 1.4	12.6 ± 1.4	12.5 ± 1.4	12.5 ± 1.5		
MCV (fL)	nLRWB	65.7 ± 4.3 *	61.1 ± 4.2 *	60.4 ± 4.5 *	60.9 ± 4.4 *	62.17 ± 4.9 *	63.3 ± 4.7 *	64.8 ± 5.0 *	**<0.001**	0.06
	LRWB	65.59 ± 4.31 *	60.41 ± 4.38 *	59.70 ± 4.60 *	59.97 ± 4.57 *	60.57 ± 4.88 *	61.33 ± 4.88 *	61.94 ± 5.23 *		
RDW (%)	nLRWB	13.6 ± 0.7	13.74 ± 0.7	13.67 ± 0.7	13.87 ± 0.7	14.20 ± 1.2 *	14.81 ± 1.5 *	15.77 ± 1.8 *	**0.02**	0.89
	LRWB	13.47 ± 0.76	13.58 ± 0.7	13.76 ± 0.8	14.09 ± 0.9	14.27 ± 1.1 *	14.71 ± 1.3 *	15.29 ± 1.8 *		
WBC (×10^9^/L)	nLRWB	8.87 ± 1.31	9.25 ± 1.77	9.29 ± 1.60	9.14 ± 1.79	9.22 ± 1.77	9.12 ± 1.90	8.57 ± 1.80	0.07	**<0.001**
	LRWB	0.027 ± 0.06	0.002 ± 0.00	0.00 ± 0.01	0.00 ± 0.00	0.00 ± 0.00	0.00 ± 0.00	0.00 ± 0.01		
PLT (×10^9^/L)	nLRWB	227.7 ± 97.9	209.0 ± 95.1	175.7 ± 90.2	186.4 ± 83.5	176.2 ± 80.3	165.5 ± 76.9	191.2 ± 77.9	0.08	**<0.001**
	LRWB	11.5 ± 19.6	4.1 ± 6.3	5.0 ± 8.4	6.8 ± 8.6	9.8 ± 9.8	12.2 ± 10.7	16.7 ± 11.5		

The significant results (*p* < 0.05) of the multivariable analysis are in bold. Asterisks * indicate the significant times (*p* < 0.05).

**Table 2:** Means ± standard deviations and significance of the parameters Na^+^, K^+^, and Cl^−^(mEq/L), LDH(U/L) in nLRWB and LRWB groups and in the different days post-donation.

Parameters	Type of Units	Day of Donation							*p* for Repeated Measurements	
		T0 Day of Donation	T1 Day 7	T2 Day 14	T3 Day 21	T4 Day 28	T5 Day 35	T6 Day 42	Among Different Times	Between Groups (nLRWB vs. LRWB)
**Na^+^** **(mEq/L)**	**nLRWB**	154.0 ± 1.00 *	159.20 ± 0.84	158.40 ± 7.70	164.80 ± 5.45	163.00 ± 2.35	166.00 ± 3.16 *	169.20 ± 3.56 *	**<0.001**	0.37
	**LRWB**	153.2 ± 2.4 *	158.2 ± 1.3	156.6 ± 6.1	162.0 ± 4.1	159.8 ± 2.7	161.4 ± 3.2 *	165.6 ± 3.4 *		
**K^+^** **(mEq/L)**	**nLRWB**	2.5 ± 0.2 *	3.9 ± 0.3 *	4.1 ± 0.5 *	4.3 ± 0.4 *	4.6 ± 0.4 *	4.8 ± 0.4 *	5.0 ± 0.5 *	**<0.001**	0.21
	**LRWB**	2.5 ± 0.2 *	3.8 ± 0.2 *	4.0 ± 0.3 *	4.2 ± 0.3	4.2 ± 0.4	4.2 ± 0.4 *	4.3 ± 0.4 *		
**Cl^−^** **(mEq/L)**	**nLRWB**	78.0 ± 4.1	80.8 ± 3.9 *	79.6 ± 2.5	79.2 ± 3.2	77.4 ± 2.7	77.2 ± 3.2	77.6 ± 3.1	**<0.01**	0.75
	**LRWB**	79.4 ± 5.1	81.8 ± 4.6 *	80.4 ± 2.8	82.2 ± 5.4	80.4 ± 3.5	80.0 ± 4.3	80.6 ± 4.0		
**LDH** **(U/L)**	**nLRWB**	237.4 ± 255.6	217.2 ± 76.6	201.8 ± 67.2	258.2 ± 62.8	374.0 ± 153.2 *	612.4 ± 173.5 *	808.6 ± 108.7 *	**<0.001**	**<0.001**
	**LRWB**	27.0 ± 15.3	63.4 ± 17.8	61.2 ± 35.0	69.4 ± 26.7	111.6 ± 30.9 *	129.4 ± 39.5 *	162.2 ± 61.3 *		

The significant results of the multivariable analysis are in bold. Asterisks * indicate the significant times (*p* < 0.05).

## Data Availability

Not applicable.
